# Immunogenicity of porcine circovirus type 2 nucleic acid vaccine containing CpG motif for mice

**DOI:** 10.1186/s12985-016-0597-0

**Published:** 2016-11-14

**Authors:** Jun Li, Jiang Yu, Shaojian Xu, Jianli Shi, Shengnan Xu, Xiaoyan Wu, Fang Fu, Zhe Peng, Lingling Zhang, Shuxuan Zheng, Xiaoyuan Yuan, Xiaoyan Cong, Wenbo Sun, Kaihui Cheng, Yijun Du, Jiaqiang Wu, Jinbao Wang

**Affiliations:** 1 Division of Swine Diseases, Shandong Provincial Key Laboratory of Animal Disease Control & Breeding, Institute of Animal Science and Veterinary Medicine Shandong Academy of Agricultural Sciences, Jinan, 250100 China; 2 Division of Swine Infectious Diseases, State Key Laboratory of Veterinary Biotechnology, Harbin Veterinary Research Institute, Chinese Academy of Agricultural Sciences, Harbin, 150001 China

**Keywords:** Porcine circovirus type 2, Nucleic acid vaccine, Immunogenicity, CpG motifs, Mice

## Abstract

**Background:**

This study aimed at reseaching the immune effect of porcine circovirus type 2 (PCV2) DNA vaccine containing CpG motif on mice.

**Methods:**

A total of 40 6-week-old female BALB/c mice were randomly divided into four groups which were immunized by 18CpG-pVAX1-ORF2, pVAX1-ORF2, pVAX1 and PBS, respectively, and immunized again 2 weeks later. All mice were challenged with 0.2 mL PCV2 cells virulent strain SD (10^6.0^ TCID_50_/mL) after 4 weeks. Average daily gain, blood antibody levels, microscopic changes and viremia were detected to estimate the effect of DNA vaccine.

**Results and Discussion:**

The results showed that compared to those of the control mice, groups immunized with pVAX1-ORF2 and 18CpG-pVAX1-ORF2 could induce PCV2-specific antibodies. The PCV2-specific antibodies level of 18 CpG-pVAX1-ORF2 groups was higher significantly than other groups and decreased slowly along with time. There was no distinct pathological damage and viremia occurring in mice that inoculated with CpG motif DNA vaccines. The results demonstrated that the DNA vaccine containing 18 CpG could build up resistibility immunity and reduce immune organ damage on mice.

## Background

Porcine circovirus (PCV) is a small, nonenveloped, icosahedral virus containing a circular single-stranded DNA genome, which is assigned to the Circoviridae family. PCV comprises with two genotypes, which are non-pathogenic PCV1 and pathogenic PCV2 [1]. The former exist widely in PK-15 cells, and the latter is closely related with postweaning multisystemic wasting syndrome (PMWS) [2], which mainly infect weaned pigs and fattening pigs. Nowadays, porcine circovirus type 2 (PCV2) has become one of the most important pathogens affecting the swine industry worldwide [3]. PCV2 contains at least two major open reading frames (ORFs). ORF1 encodes the replication proteins (Rep and Rep’) which involved in virus replication, and ORF2 encodes the capsid proteins (Cap) were found to be immunogenic which made them suitable for vaccine development [4].

Currently, PCV2 vaccination is still an important method to combat porcine circovirus diseases (PCVD). At present, chimeric viruses, subunit vaccines, recombinant vaccines, genetic engineering vaccines and other kind of vaccines were researched at home and abroad. However, the most successful vaccine candidates were those based on the induction of an active immune response against the capsid protein of PCV2 [5–7].

Some domestic and international reports have showed that CpG motifs have the effect of immunostimulation as an immune adjuvant [8, 9]. CpG motifs can activate the immune system by enhancing the antigen presentation capacity of APC. The current research on CpG as immune adjuvant was mainly focused on the mouse and human disease. Based on the earlier research in the authors ‘laboratory (Cheng KH, “Study on Series of DNA Vaccines Against Porcine Circovirus Type 2[D],”[master’s thesis QingDao Agricultural University, 2009]), we first reported the CpG motifs as an adjuvant insert to the PCV2 DNA vaccine that could boost immunity in pigs [10].

Our research and other research had showed that mouse could be infected by PCV2 and used as a PCV2 infected experimental model [11, 12]. In this study,we evaluate immune effect of PCV2 DNA vaccine with CpG motif on mice using the best CpG motif from our earlier research [10], which can provide a great prospect for preventing and controlling PCVD,in order to give candidate vaccine evaluation model.

## Methods

### Viruses and vaccine

The titer of PCV2 strain SD on PK-15 cells (DQ478947) was 10^6.0^ TCID_50_/ml. PCV2 strain SD,DNA vaccine plasmid 18CpG-pVAX1-ORF2,plasmids pVAX1-ORF2 and pVAX1 were constructed by Cheng Kaihui and saved by Shandong Key Laboratory of Animal Disease Control & Breeding. PCV2 strain SD was also preserved in Chinese bacterium Preservation Center (CGMCC NO.5774).

### Animal vaccination

Six-week old female BALB/c mice (Shandong province Experimental Animal Centre, China) were divided randomly into four groups (10 mice/group), which were immunized intramuscular injection in legs by 18CpG-pVAX1-ORF2, pVAX1-ORF2, pVAX1 or PBS, respectively, and immunized again after 2 weeks (Table 1). All mice were challenged intramuscularly with 0.2 mL PCV2 cells virulent strain SD (10^6.0^ TCID_50_/mL) after 4 weeks. Average daily gain was recorded everyday during the experiment. All mice experimental procedures were performed in accordance with the Regulations for the Administration of Affairs Concerning Experimental Animals approved by the State Council of People’s Republic of China.

**Table 1:** Groups distribution and Immunization of six-week-old mice

Groups	Immunized recombinant plasmid	Dosage
I(Control)	PBS	0.2 mL
II	pVAX1	0.2 mL(Plasmid concentration 500 μg/mL)
III	pVAX1-ORF2	0.2 mL(Plasmid concentration 500 μg/mL)
IV	18CpG-pVAX1-ORF2	0.2 mL(Plasmid concentration 500 μg/mL)

### Physical signs studies

The change of body weight was recorded at the time of vaccinations, and before PCV2 cells virulent strain challenge. Average daily gain was calculated to evaluate the vaccine effection.

### Assay of mice blood antibody levels

The blood samples was collected at the time of vaccinations, 2 weekly intervals during immunity period and weekly after challenge until necropsy, respectively. The serum were separated and detected blood antibody levels with PCV2-dCap-ELISA kit (Tianjin ringpu biotechnology Limited by Share Ltd) according to the manufacturer’s directions. The positive cutoff was set at S/P ≥ 0.25 when S/P = (OD_450_ of sample - OD_450_ mean of negative control)/(OD_450_ mean of positive control - OD_450_ mean of negative control).

### Pathological and histopathology studies

The mice were euthanized at 3 weeks after challenge and samples from the lung, spleen and liver were fixed in 10 % neutral-buffered formalin solution, and were sectioned and stained with hematoxylin and eosin (HE). Microscopic changes were determined by comparing the tissues of the challenged mice with those of the control-groups.

### Assay of PCV2 distribution by Real-time PCR

Real-time PCR was used to detect the virus in the neck muscles, heart, liver, spleen, lung, kidney and blood. One pair of polymerase chain reaction (PCR) primers D1 5′-TTACCGGCGCACTTCGGCAG -3′ and D2 5′-ACTCCGTTGTCCCTGAGAT -3′ were designed using computer software (Primer Premier 5.0) according to the published sequence of the PCV2 in GenBank (DQ478947) to establish a fluorescent quantitative PCR assay for the detection of PCV2 (404 bp). The amplification was carried out in a 25 μL reaction containing 1 μM of each forward and reverse primer, 12.5 μL of SYBR Green I Mix (TaKaRa), 2 μL DNA templates, and ddH_2_O up to 25 μL. The PCR conditions were as follows: 95 °C for 10 min, followed by 30 cycles of amplification at 95 °C for 10 s, and 55°Cfor 10 s [13]. The assays were repeated at least three times, with each experiment performed in triplicate.

### Statistical analysis

All data were performed using SPSS 15.0 software (SPSS Inc., Chicago, IL, USA). Statistical analyses were analyzed by two-way analysis of variance (ANOVA) followed by Tukey’s post hoctest, respectively. Differences were considered significance at *p* < 0.05.

## Result

### Average daily gain (ADG)

Body weight was recorded at day 1 and 4 week, ADG of every mouse was calculated and statistical analyses were performed. The result showed that compared to the control mice (group I),there was no significant difference among groups (*p* ≥ 0.05), and the weight gain was not altered by vaccination in mice.

### Assay of blood antibody detection

**Fig. 1:**
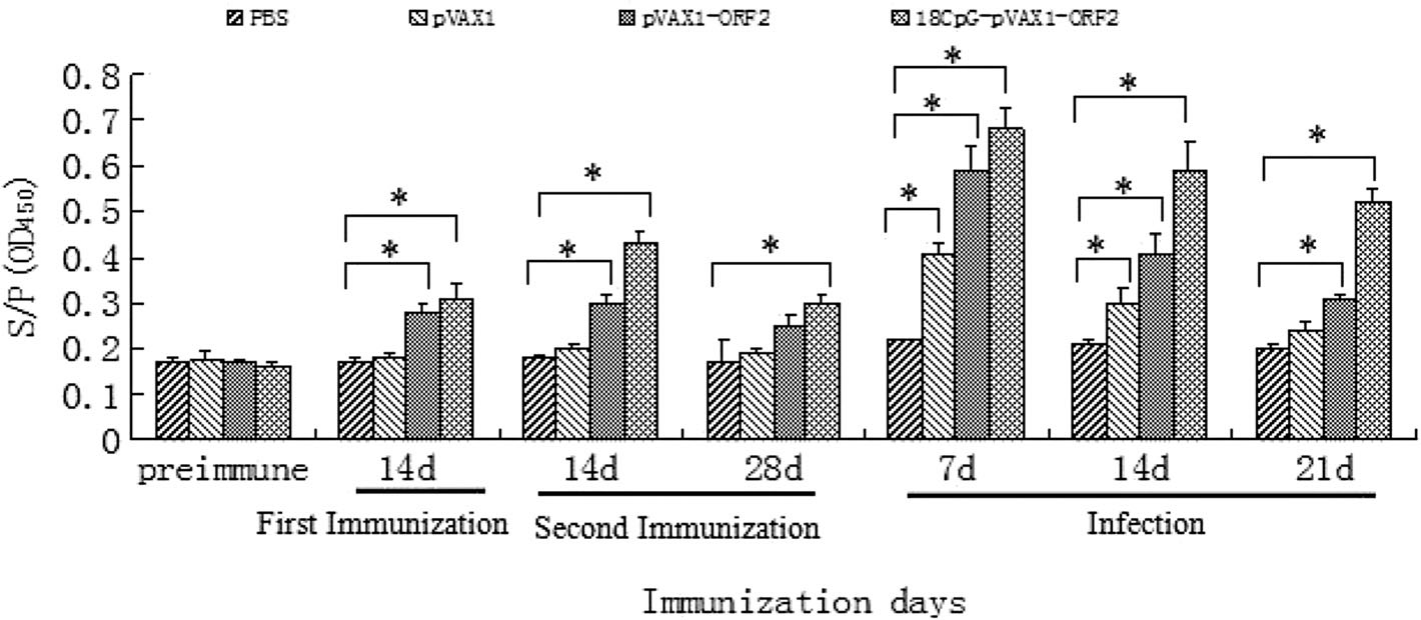
Serum antibody level after immunization in mice

Porcine circovirus type 2 (PCV2)-antibody responses monitored by PCV2-ORF2 protein enzyme-linked immunosorbent assay. The antibody level of group III and IV was rised gradually after first immunization, and group IV showed an obvious rise after secondary immunization, while group III rised within a narrow range. However, the antibody of group IV was still up to 0.3097 at 4 week after secondary immunization, which was positive. Thus, the immune effect of DNA vaccine containing 18 CpG was better than other groups throughout the time after vaccination. After challenge with PCV2 strain SD, the antibody level of the four groups was all rised due to the anamnestic response in the vaccinated groups, and the highest level was appeared at 1 week after challenge. The antibody level of group IV was higher significantly than other groups (*p* < 0.05), and decreased slowly along with time (Fig. 1). The results of mice blood antibody detection (S/P) was showed in Table 2.

**Table 2:** Serum antibody level after immunization in mice (S/P)

Groups	Pre-immune blood	Blood after the first immunization two weeks	Blood after the second immunization two weeks	Blood after the second immunization four weeks	Blood after injection one week	Blood after injection two weeks	Blood after injection three weeks
I	0.1507	0.1510	0.1601	0.1500	0.2329	0.2206	0.1993
II	0.1507	0.1628	0.2052	0.1862	0.4249	0.3084	0.2426
III	0.1507	0.2576	0.3003	0.2417	0.5928	0.4267	0.3223
IV	0.1507	0.3127	0.4340	0.3097	0.6754	0.5795	0.5186

### Clinical observation and pathological changes

All mice of group I and II becoming lean, almost emaciated and displayed splenomegaly, No obvious clinical signs and gross lesions were observed in the vaccinated group mice of group III and IV.

**Fig. 2:**
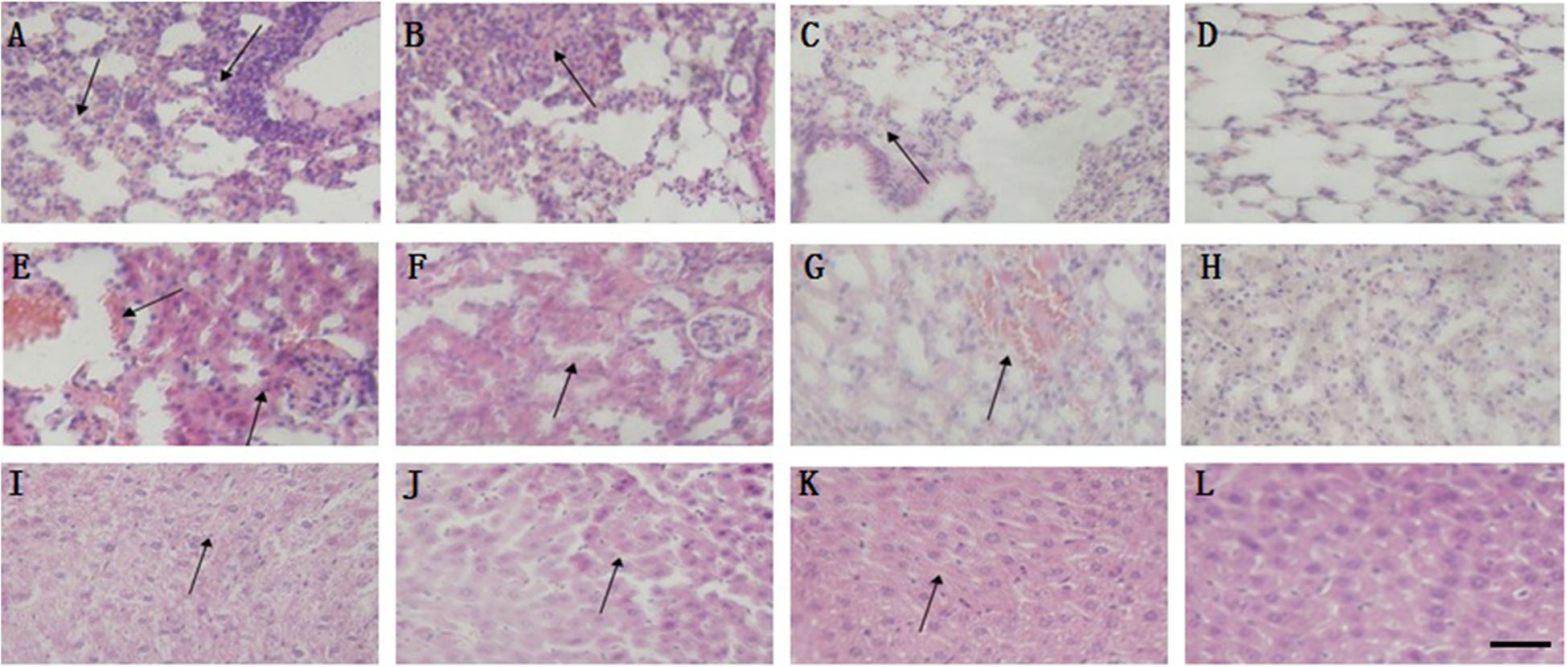
Histopathological changes in the lungs (A-D), kidney (E-H) and liver (I-L) of mice infected by PCV2 (HE). Mice of group I (PBS) showed alveolar walls were thick and inflammatory cells were in the lung bronchia (**a**). Mice of group II (pVAX1) showed interstitial pneumonia, and congestion was observed in alveolar interlobular interval and infiltration with inflammatory cells (**b**). A few of inflammatory cells were observed in alveoli of group III (pVAX1-ORF2) (**c**). Wider congestion in the glomeruli was observed in group I-III, and degenerative necrosis in kidney tubules were observed in group I and II (**e**, **f**). The typical or slight vacuoles granular degeneration was observed in hepatocyte of group I or III, respectively (**i**, **k**). The hepatocyte of group II showed slight atrophy (**j**). No obvious unusual changes were observed in lungs, liver and kidney of group IV (**d**, **h**, **l**). The scale bar is 100 mm

The alveolar walls thicked and inflammatory cells infiltrated in lung bronchia were showed in mice of group I (Fig. 2a). Interstitial pneumonia, and congestion were observed in alveolar interlobular interval and infiltration with inflammatory cells (Fig. 2b) while the hepatocyte showed slight atrophy and hemorrhage (Fig. 2j) in group II mice. A few of inflammatory cells were observed in alveoli of group III (Fig. 2c). Degenerative necrosis in kidney tubules and wider congestion in the glomeruli were observed in group I-III (Fig. 2e, f, g). Typical slight vacuoles granular degeneration was observed in hepatocyte of group I (Fig. 2i) and III (Fig. 2k), respectively. No obvious unusual changes were observed in lungs, kidney and liver of group IV (Fig. 2d, h, l). The result showed that the DNA vaccine containing 18 CpG could provide an effective immune protection.

### Quantitative analysis of PCV2 expression in tissues and blood

**Fig. 3:**
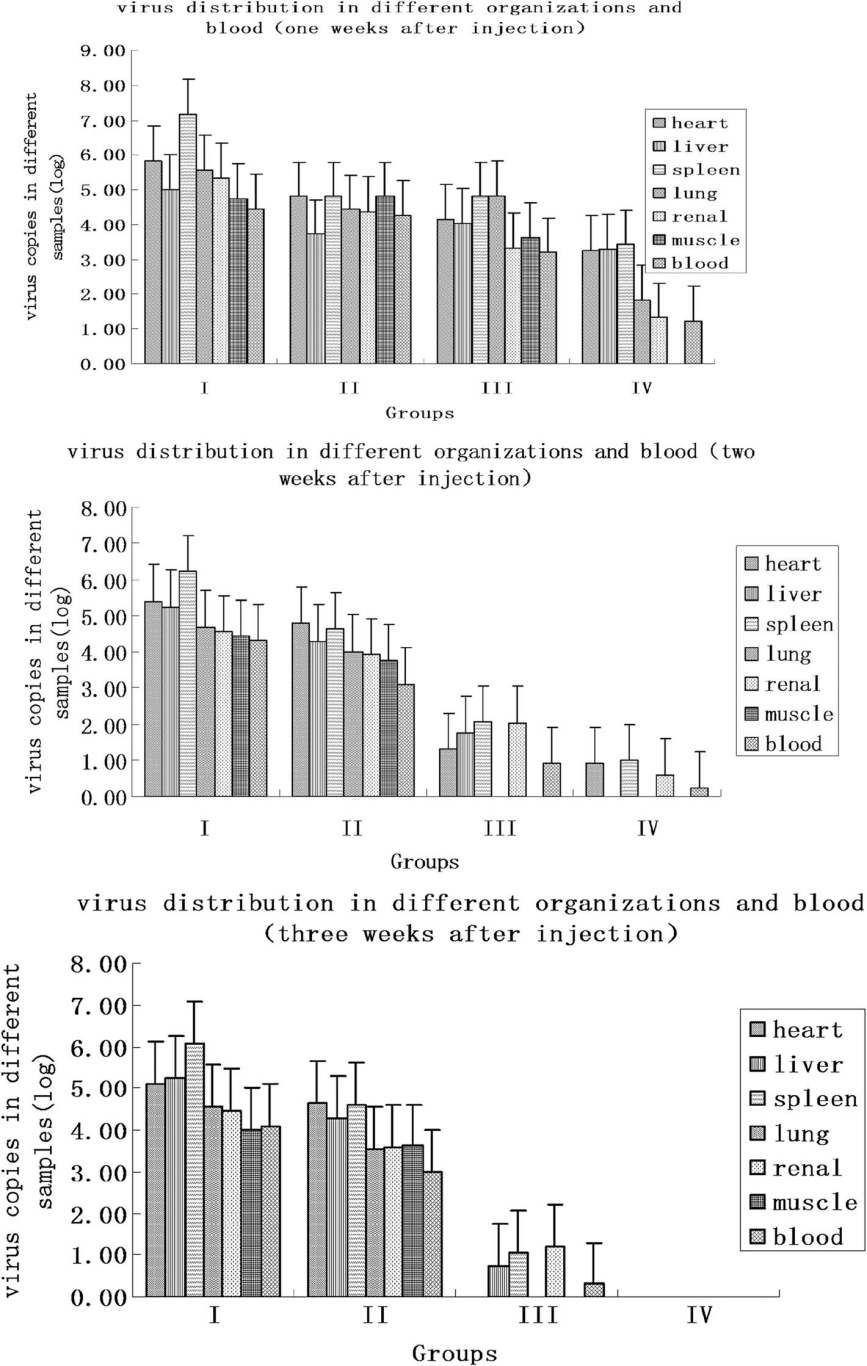
The distribution of virus in different organizations of different periods

The PCV2 contents in blood and different tissues were calculated at 1, 2 and 3 weeks after injection (Table 3, Fig. 3). The results showed that PCV2 were not detected in the position of injection which is neck muscles. Atthe first week after injection, while the PCV2 contents of groups III and IV were less than groups I and II (*p* < 0.05). PCV2 were not detected in the tissues and blood of group IV at 3 weeks after injection, while the liver, spleen, kidney and blood have a few PCV2, which explained the virus was not eliminated completely. In addition, the amount of PCV2 in spleen was the highest in all the tissues and blood (Fig. 2).

**Table 3:** The testing result of PCV2 in the organs of mice

days	Groups	heart	liver	spleen	lung	renal	muscle	blood
7d	I	+	+	+	+	+	+	+
	II	+	+	+	+	+	+	+
	III	+	+	+	-	+	+	+
	IV	+	+	+	+	+	-	+
14d	I	+	+	+	+	+	+	+
	II	+	+	+	+	+	+	+
	III	+	+	+	-	+	-	+
	IV	+	-	+	-	+	-	+
21d	I	+	+	+	+	+	+	+
	II	+	+	+	+	+	+	+
	III	-	+	+	-	+	-	+
	IV	-	-	-	-	-	-	-

## Discussions

PCV2, the primary causative agent of PMWS, PRDC, mainly infected the weaned piglets (Allan et al., 1998), resulting in substantial economic losses to the pig industry. At present, there were not good drugs to treat with the diseases caused by PCV2. Thus, vaccine immunization has become a fundamental means and measures to prevent PCV2.

Nucleic acid vaccine is one of the hot spot of PCV2 vaccine researches. DNA vaccines may be capable of inducing immunity regardless of maternally derived antibodies [14, 15] and they have induced protective cellular and humoral immunity in preclinical models of infectious diseases. PCV2 Nucleic acid vaccine was first reported by Kamstrup [16], they developed and investigated the potential of DNA vaccination approach to against PCV2. Mice were vaccinated three times by gene gun and all mice responded serologically by raising antibodies against PCV2. They found that vaccination based on DNA might offer opportunities for vaccination of piglets against PCV2.

However, DNA vaccine applications are limited due to the problems related to delivery, species of the immunized animals and degradation of plasmid DNA. To compensate for these limitations, numerous studies have explored methods to improve immune responses induced by DNA immunization by optimizing plasmid design, vaccine delivery systems and adjuvants [17]. Adjuvants are of particular interest because they may enhance DNA delivery and increase the magnitude and duration of plasmid DNA expression [18]. Guo (2015) reported the enhancement of the immunogenicity of porcine circovirus type 2 DNA vaccines by using a recombinant plasmid co-expressing capsid protein and porcine interleukin-6 in mice. Dong [19] found that the mice co-inoculated with pVAX-PCV2-ORF2 plus pVAX-pIL-15 have higher humoral and cellular immune responses than the others. In addition, DNA plasmid bearing PCV2 ORF2 gene has a protective effect against challenge with PCV2 in mice which could be promoted with the utilization of pIL-15.Fu [20] evaluated three adjuvants Ubiquitin (ub), the peptide binding truncated C-terminal portion of heat shock protein 70 (hsp70c) and interleukin-2 (IL-2) in PCV2 DNA vaccine, they found that ub is a superior adjuvant for PCV2 DNA vaccination than the hsp70c and IL-2 molecules. Chen [21] evaluated two recombinant plasmids containing the ORF2 gene of porcine circovirus type 2 (PCV2) with or without porcine interleukin-18 (IL-18) and found that the plasmid pBudCE4.1-ORF2/IL18 may be an effective approach for increasing the immunogenicity of PCV2 DNA vaccine.

Our previous research demonstrated that CpG motifs as an adjuvant could boost the humoral and cellular immunity of pigs to against PCV2, especially in terms of cellular immunity [10]. This study evaluated the adjuvant of CpG motifs to PCV2 DNA vaccine and mainly detected the serum antibody levels in mice by ELISA. The results showed that the antibody levels of immune groups were increased, especially the nucleic acid vaccine containing 18 CpG, kept at a higher level throughout the immunization and decreased slowly, after challenge, it increased rapidly and could maintain a longer time.

The result of quantitative real-time PCR showed that PCV2 was replicated in the tissues of mice, while it was eliminated gradually in immune groups. Moreover, the content of PVC2 in spleen of all the tissues was the highest. The result of autopsy declared obvious lesions were appeared in group I and II, and minimal lesion was appeared in group III. No obvious unusual changes were observed in group IV.

## Conclusions

In conclusion, the nucleic acid vaccine containing 18 CpG can build up resistibility immunity and reduce immune organ damage on mice. which has a wide prospect for preventing and controlling PCV2 infection.
